# Dipalmitoylphosphatidylcholine Lipid Vesicles for Delivering HMB, NMN, and L-Leucine in Sarcopenia Therapy

**DOI:** 10.3390/molecules30071437

**Published:** 2025-03-24

**Authors:** Alfred Najm, Alexandra Cătălina Bîrcă, Adelina-Gabriela Niculescu, Adina Alberts, Alexandru Mihai Grumezescu, Bianca Gălățeanu, Bogdan Ștefan Vasile, Mircea Beuran, Bogdan Severus Gaspar, Ariana Hudiță

**Affiliations:** 1 Carol Davila University of Medicine and Pharmacy, 8 Eroii Sanitari, Sector 5, 050474 Bucharest, Romania; alfred.najm@yahoo.ro (A.N.); adina-magdalena.alberts@rez.umfcd.ro (A.A.); drmirceabeuran@yahoo.com (M.B.); bogdangaspar2005@yahoo.com (B.S.G.); 2 Emergency Hospital Floreasca Bucharest, 8 Calea Floreasca, Sector 1, 014461 Bucharest, Romania; 3 National University of Science and Technology Politehnica Bucharest, 011061 Bucharest, Romania; ada_birca@yahoo.com (A.C.B.); adelina.niculescu@upb.ro (A.-G.N.); bogdan.vasile@upb.ro (B.Ș.V.); 4 Research Institute of the University of Bucharest—ICUB, University of Bucharest, 050657 Bucharest, Romania; bianca.galateanu@bio.unibuc.ro (B.G.); ariana.hudita@bio.unibuc.ro (A.H.); 5 Faculty of Biology, University of Bucharest, 050657 Bucharest, Romania

**Keywords:** DPPC, lipid vesicles, sarcopenia, increased NAD+ levels, NMN, HMB, L-Leucine

## Abstract

Sarcopenia, characterized by the degeneration of skeletal muscle tissue, has emerged as a significant concern in recent years. This increased awareness stems from advances in research focusing on elderly patients, which have revealed correlations between aging mechanisms and muscle degeneration, beyond the mere fact that tissues age and deteriorate over time. Consequently, the present study aims to address sarcopenia by developing and evaluating DPPC lipid vesicles that encapsulate three distinct drugs: HMB, NMN, and L-Leucine. These drugs are specifically selected for their properties, which facilitate effective interaction with the affected muscle tissue, thereby promoting desired therapeutic effects. Preliminary physicochemical analyses indicate the successful formation of spherical lipid vesicles, characterized by nanometric dimensions and stable membrane integrity. The biological investigations aimed to highlight the potential of DPPC lipid vesicles encapsulating HMB, NMN, and L-Leucine to alleviate sarcopenia-induced cytotoxicity and oxidative stress. Through a comparative evaluation of the three drug formulations, we demonstrate that drug-loaded DPPC vesicles effectively mitigate oxidative damage, preserve mitochondrial function, and maintain cytoskeletal integrity in H_2_O_2_-induced C2C12 myotubes, with HMB-loaded vesicles showing the strongest protective effects against muscle degeneration. These findings underscore the therapeutic potential of DPPC-based controlled release systems for sarcopenia treatment and highlight the need for further investigations into their mechanistic role in muscle preservation.

## 1. Introduction

Human tissues undergo significant changes over the course of a lifetime, particularly in older individuals, who frequently confront issues related to tissue degradation. For instance, after the age of 50, muscle mass typically begins to decrease annually at a rate of 1 to 2%. This progressive loss of muscle is known as sarcopenia. While it was once primarily linked to aging and tissue deterioration, sarcopenia is now recognized as being influenced by a variety of factors, including chronic diseases, malnutrition, reduced mobility, and a sedentary lifestyle [1,2,3]. Sarcopenia can be categorized into two distinct types. Primary sarcopenia primarily results from aging, leading to the weakening of muscle fibers and a decrease in the number of motor neurons. In contrast, secondary sarcopenia is driven by factors such as physical inactivity, malnutrition, certain medications, and various chronic diseases [4]. To accurately diagnose sarcopenia while accounting for age-related variations in muscle mass, the European Working Group on Sarcopenia in Older People (EWGSOP) recommends a four-step diagnostic algorithm known as the F-A-C-S algorithm. The name reflects the key activities involved in the process: “Find” (identify potential cases), “Assess” (evaluate grip strength), “Confirm” (determine muscle quantity and quality), and “Severity” (assess muscle performance to gauge the extent of the condition) [5].

Sarcopenia is particularly prevalent among the elderly population, with estimates suggesting it affects 10% to 16% of older adults worldwide. This condition significantly increases the risk of various health issues, including bone fractures, osteoporosis, obesity, cardiovascular disease, insulin resistance, type II diabetes, chronic inflammation, and, ultimately, mortality [5,6,7]. However, there are other diseases associated with the risk of sarcopenia, such as respiratory diseases, heart disease, anorexia, cognitive impairment, depression, and Parkinson’s [7].

The loss of skeletal muscle mass that occurs with aging is primarily attributed to a reduction in the number of myofibers, coupled with the atrophy of individual myofibers [8]. In cases of secondary sarcopenia—often resulting from prolonged bed rest or physical inactivity—the loss is mainly due to a decrease in the cross-sectional area of the myofibers. Myofibers, which are multinucleated cells formed by the fusion of satellite cells, constitute the fundamental structure of skeletal muscles. Satellite cells are specific to skeletal muscle and play a crucial role in muscle regeneration; they are often referred to as somatic stem cells. While skeletal muscles can sustain damage from trauma or overuse, their cellular structure allows for significant regenerative capacity. This regenerative ability is particularly notable in younger individuals and those without accompanying health conditions [9,10,11]. Skeletal muscles are primarily maintained, regenerated, and enhanced through physical exercise. However, many patients find it challenging to engage in various muscle regeneration exercises. As a result, the pharmaceutical sector plays a crucial role in addressing this issue through drug therapy [12]. To effectively implement drug therapy for sarcopenia, it is essential to consider both intrinsic and extrinsic factors. Intrinsic factors include inflammation, mitochondrial function, autophagy, apoptosis, calcium metabolism, and neuromuscular junction integrity. Extrinsic factors encompass nutritional status, impaired mobility, and hormonal influences. Recognizing and addressing these factors is key to developing effective treatment strategies for sarcopenia [11,13]. Despite the identification of various associated factors, the exact mechanisms underlying the induction of sarcopenia remain unclear. However, mitochondrial dysfunction is increasingly recognized as a central pathogenetic factor. Mitochondria are crucial for maintaining skeletal muscle health, performing multiple roles in cellular processes. Their primary function is energy production, but they are also involved in regulating cellular proliferation, coordinating intracellular calcium homeostasis, and managing apoptotic signaling. Mitochondria are essential for proper cellular functioning and maintaining homeostasis. When mitochondrial activity is compromised, it can disrupt these vital processes and lead to muscle degeneration. Research using laboratory animal models has shown that the abnormal accumulation of mitochondrial DNA (mtDNA) is a key contributor to mitochondrial dysfunction. This dysfunction results in numerous age-related issues, including the early onset of sarcopenia. The abnormal accumulation of mtDNA is directly correlated with various dysfunctions that promote the production of mitochondrial reactive oxygen species (ROS) in aging muscle and neuronal tissues. These processes underscore the critical role of mitochondrial health in the development of sarcopenia and other age-related phenotypes [14,15,16,17,18].

Progressive skeletal muscle loss results from the dysregulation of various physiological processes, yet effective therapies remain elusive. Nicotinamide adenine dinucleotide (NAD+) is a crucial cofactor and signaling mediator in many enzymatic reactions. NAD+ deficiency contributes to tissue degeneration by disrupting redox metabolism, mitochondrial quality, cell cycle, autophagy, cellular stress responses, ionic homeostasis, and circadian rhythms. Preclinical studies indicate that NAD+ deficiency leads to skeletal muscle degeneration and premature death due to bioenergetic deficits, mitochondrial dysfunction, calcium dysregulation, inflammation, senescence, and depletion of myosatellite cells. Restoring NAD+ levels has been shown to improve muscle homeostasis [19,20,21,22].

Given this information, a pharmacological approach is essential for treating sarcopenia, particularly considering that many individuals affected by this condition are unable to engage in physical exercises known to enhance muscle tissue. Therefore, a key objective of research in this field is to develop an anti-sarcopenia medication that targets the maintenance and improvement of muscle function from both pharmacological and chemical perspectives. This innovative treatment could provide a viable alternative for those unable to participate in traditional exercise regimens, ultimately contributing to better health outcomes for patients suffering from sarcopenia [23].

**Figure 1:**
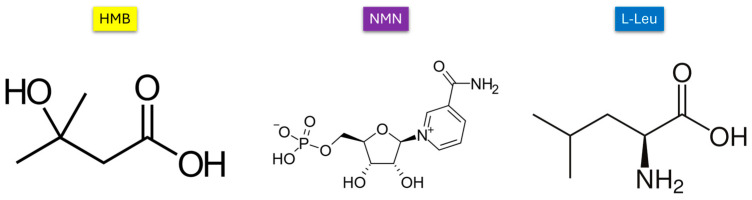
Chemical structures of HMB, NMN, and L-Leucine.

In this study, we develop and test a drug-delivery system designed to enhance muscle function through the use of drug-loaded vesicles. These vesicles are self-assembled structures that can be cultivated in the laboratory. They consist of one or more bilayers, creating a central aqueous environment within their structure. This innovative approach aims to optimize the delivery of therapeutic agents directly to muscle tissues, potentially improving their effectiveness and overall impact on muscle health [24,25,26,27]. An amphiphilic compound, dipalmitoylphosphatidylcholine (DPPC), was utilized due to its ability to self-aggregate in aqueous environments and form lipid bilayers. When the dispersion is heated above its melting transition temperature of 41 °C, DPPC creates fluid liposomes. Upon cooling this dispersion to 25 °C or 37 °C, frozen liposomes or vesicles are formed. The selection of DPPC offers a significant advantage due to its structural and compositional resemblance to biological membranes, enhancing its potential for effective integration within biological systems [28,29,30]. As a result, we developed a liposome-based support system capable of encapsulating three distinct active substances: beta-hydroxy-beta-methylbutyrate (HMB), nicotinamide mononucleotide (NMN), and L-Leucine (L-Leu), whose chemical structures are represented in Figure 1. Each of these active compounds plays a unique role in promoting the health of the affected tissue.

HMB is an active metabolite of leucine that plays a crucial role in muscle metabolism. It works by reducing muscle protein degradation, thereby promoting an increase in muscle mass. Additionally, HMB stimulates protein synthesis and helps diminish reactive oxygen species (ROS), contributing to overall muscle health and performance [31,32,33,34,35,36]. NMN serves as a direct precursor to NAD+, enhancing NAD+ levels, which in turn improves mitochondrial function and reduces oxidative stress. This multi-faceted approach aims to provide comprehensive support for tissue recovery and performance enhancement [37,38,39,40,41,42,43]. L-Leu is an essential amino acid that helps to minimize muscle breakdown and promotes muscle protein synthesis. It also enhances energy and glucose metabolism, reduces reactive oxygen species (ROS), and supports muscle regeneration. By playing these vital roles, L-Leu contributes significantly to overall muscle health and recovery [44,45,46,47,48]. Using an in vitro sarcopenia model, a comparative evaluation of the three drug DPPC formulations was conducted, assessing their cytoprotective effects and ability to restore cellular homeostasis, with a particular focus on their role in mitigating mitochondrial dysfunction and oxidative damage.

## 2. Results

The investigation of liposome-type vesicles featuring a DPPC shell and an active core composed of selected substances was conducted from a physicochemical perspective. Subsequently, these vesicles were evaluated biologically to assess their ability to fulfill the primary functions and activities targeted in this study. Characterization was carried out on the DPPC control sample (DPPC_Ctrl) as well as on DPPC vesicles encapsulating HMB (DPPC_HMB), NMN (DPPC_NMN), and L-Leucine (DPPC_L-Leucine) at three different concentrations (4 mg, 20 mg, and 40 mg). This approach enabled a comparative analysis of their properties.

**Figure 2:**
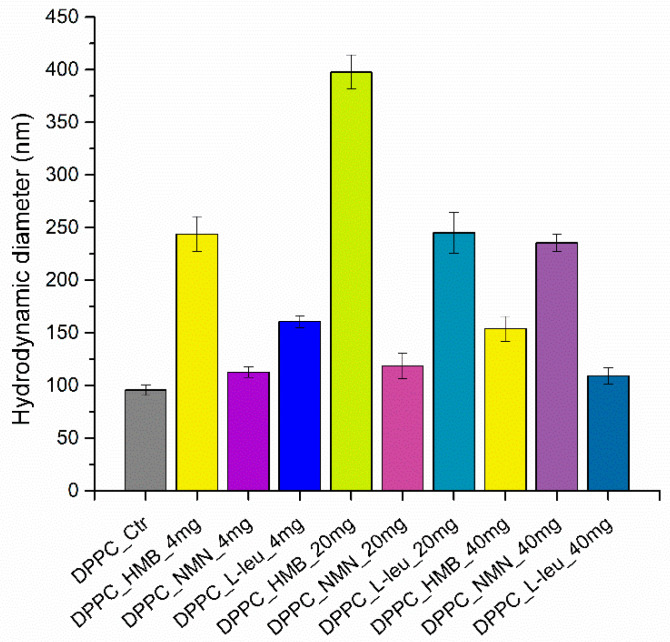
Graphic representation of hydrodynamic diameter (nm) as a DLS result of DPPC vesicles dispersed in water.

The initial analysis performed was dynamic light scattering, which is essential for assessing both the hydrodynamic diameter of the samples and their stability in liquid. Determining these two properties is critical for the effective development of drug delivery systems. Figure 2 provides the DLS result expressed as hydrodynamic diameters for all the sarcopenia-treatment vesicles.

The graphical representation of the results reveals a clear variation in the hydrodynamic diameter values based on the type of drug used. At the initial concentrations of 4 and 20 mg, the vesicles with HMB cores exhibit the highest hydrodynamic diameter, while the lowest value is associated with the DPPC encapsulating NMN at both the initial and mid-range concentrations. However, when the concentration increases to 40 mg, DPPC_HMB_40mg shows a decrease in hydrodynamic diameter, whereas DPPC_NMN_40mg displays an increase. Additionally, the behavior of the DPPC vesicles containing L-Leucine mirrors that of DPPC_HMB; for the first two concentrations, the hydrodynamic diameter increases with drug concentration, but it subsequently decreases at the maximum concentration of L-Leucine.

This trend is further illustrated in Table 1, which presents the numerical values. Both DPPC_HMB and DPPC_L-Leucine exhibit a similar pattern in terms of their hydrodynamic diameter changes in relation to drug concentration. In contrast, DPPC_NMN shows only minor variations in hydrodynamic diameter at the first two drug concentrations, but a nearly doubled measurement is observed for the DPPC_NMN_40mg sample. Additionally, the hydrodynamic diameter values range from 112 to 397 nanometers. This is based on the different interactions of DPPC with the three types of drugs incorporated into lipid vesicles, while the vesicle formation process can lead to heterogeneity in terms of hydrodynamic diameter.

**Figure 3:**
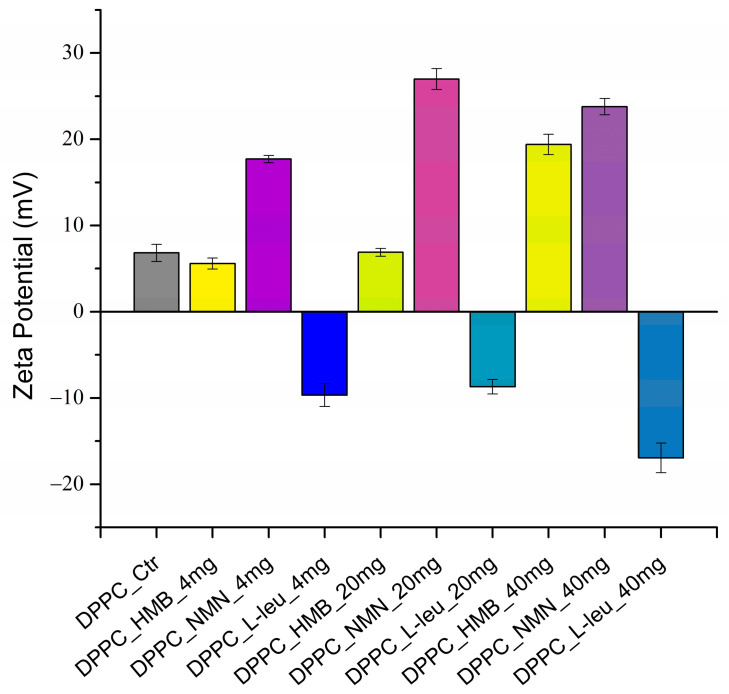
Graphic representation of zeta potential (mV) as a DLS result of DPPC vesicles dispersed in water.

The zeta potential for all obtained samples was also examined, with the results presented in Figure 3.

In the context of the stability of DPPC-based vesicles in liquid, moderate zeta potential values are noted for samples containing HMB and L-Leucine at low and medium concentrations. Notably, when L-Leucine is used as the core of the lipid vesicles, the zeta potential values exhibit a negative charge. In contrast, DPPC vesicles containing NMN display enhanced stability in liquid across all three concentrations tested. However, the zeta potential values are favorable as the drug concentration rises in the case of all the loaded DPPC vesicles. This indicates that the concentration of drug significantly influences the stability of lipid vesicles in liquid. To clearly illustrate the zeta potential values related to the stability of the samples, these values are presented in Table 2.

Table 3 presents the PDI values of each sample as well as the positioning of these values in the characteristic range.

Analyzing the PDI values resulting from the DLS analysis, it can be observed that the obtained formulations present low PDI, which coincides with good stability and consistency in terms of particle size distribution. Apart from the values of DPPC_NMN_4mg and DPPC_NMN_20mg, which fall within moderate but acceptable polydispersity, all other samples are uniform, presenting high monodisperse characteristics based on a PDI < 0.1. This is favorable and suitable for the development of lipid vesicles as a controlled drug release system type. However, this analysis result also reveals that the concentration of the drug plays a significant role. Specifically, DPPC-based vesicles with a concentration of 40 mg exhibit the best PDI values compared to those with concentrations of 20 mg and 4 mg.

**Figure 4:**
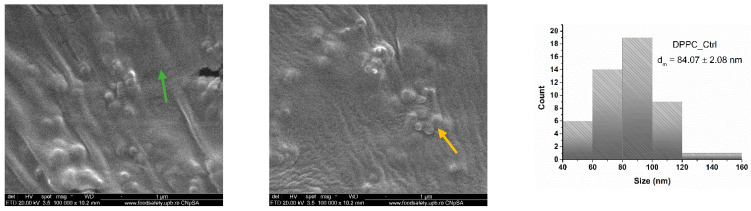
SEM micrographs and particle size distribution obtained for DPPC_Ctrl (green arrow—lipid matrix, yellow arrow—lipid vesicles).

To investigate the morphology and size of DPPC-based lipid vesicles, we conducted analyses using scanning electron microscopy (SEM). Figure 4 showcases SEM micrographs of the control sample (DPPC_Ctrl), which was analyzed without any drug incorporation. Consequently, the control sample exhibits a spherical morphology of lipid vesicles, which are dispersed within a lipid matrix that did not form vesicles. The average size of the vesicles is 84.07 ± 2.08 nm.

**Figure 5:**
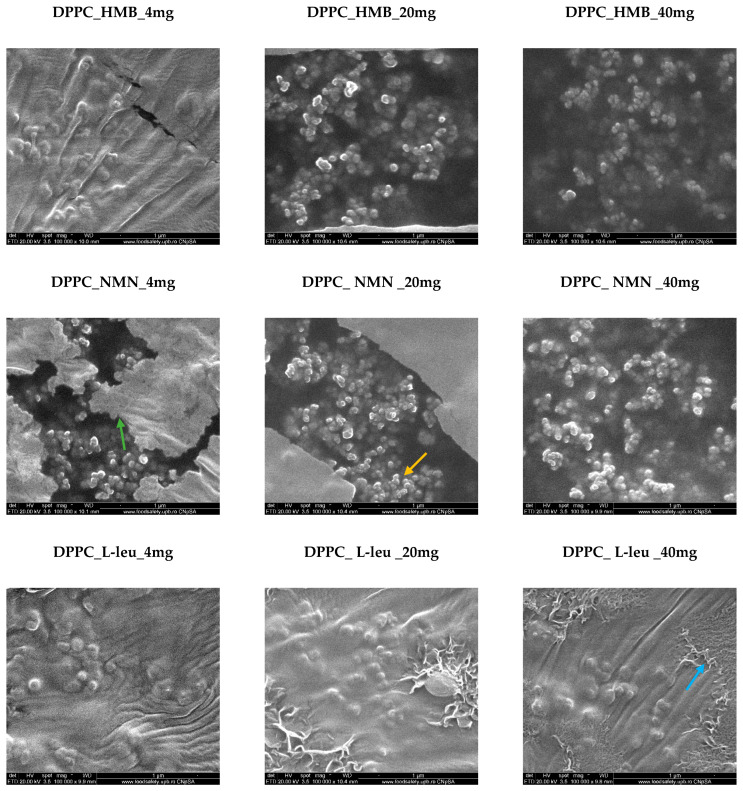
SEM micrographs obtained for DPPC_HMB, DPPC_NMN, and DPPC_L-Leucine used in three different concentrations, 4 mg, 20 mg, and 40 mg, respectively (green arrow—lipid matrix, yellow arrow—lipid vesicles, blue arrow—fibrillar structures).

The vesicles loaded with the three drugs—HMB, NMN, and L-Leucine—at varying concentrations of 4, 20, and 40 mg retain their spherical morphology (Figure 5). However, notable differences emerge in relation to the type and concentration of the drug used. As the drug concentration increases, a greater number of lipid vesicles are formed in each analyzed area. This is attributed to the absence of the lipid matrix, which did not transform into vesicles, as seen in the DPPC_Ctrl sample, allowing for a higher density of vesicles, particularly at the 20 mg and 40 mg concentrations. This observation supports the concept that the formation of lipid vesicles is enhanced when they interact with a core material to be encapsulated in an ultrasound-assisted liquid environment. In the case of DPPC, which exhibits amphiphilic properties, the three drugs interact with both the hydrophilic and hydrophobic regions of the lipid, promoting the efficient formation of vesicles according to their specific requirements. Notably, the most well-defined spherical vesicles were observed in association with the HMB and NMN drugs. Conversely, the lipid vesicle formation reaction with L-Leucine remained largely consistent across all three tested concentrations. This uniformity can be attributed to the challenges posed by the specific fibrillar structures of L-Leucine, which become more pronounced at higher concentrations, leading to increased hydrophobic interactions.

**Figure 6:**
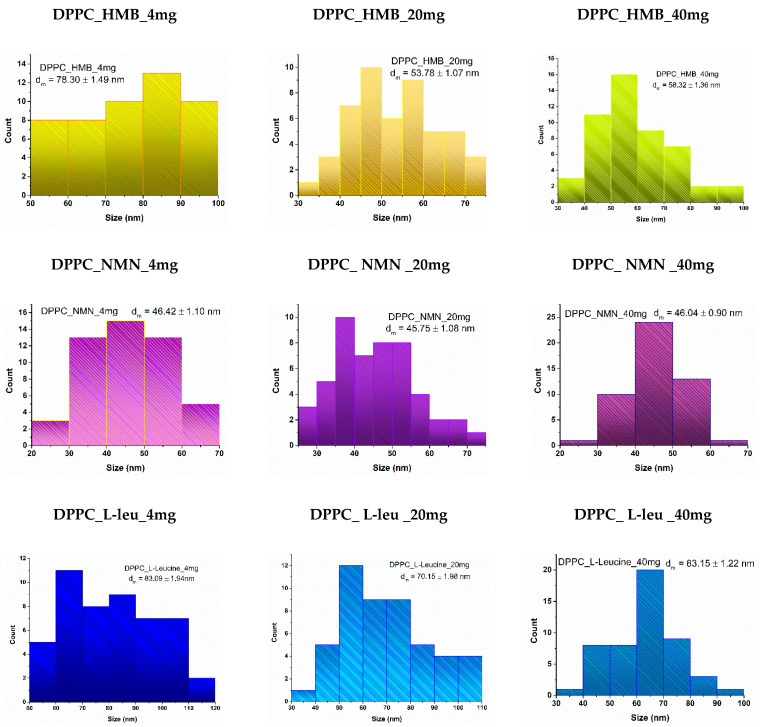
Particle size distribution obtained for DPPC_HMB, DPPC_NMN, and DPPC_L-Leucine used in three different concentrations, 4 mg, 20 mg, and 40 mg, respectively.

Lipid vesicles were analyzed to estimate their average size and to observe the variations associated with increased drug concentrations. The histograms obtained (Figure 6) for the lowest concentration of all three drug types indicated larger average vesicle sizes compared to the two higher concentrations. Notably, the vesicles fall within the nanometric range, with the smallest average sizes observed for those containing NMN across all three concentrations. Vesicles loaded with HMB exhibited intermediate sizes, while the largest average vesicle sizes were associated with the use of L-Leucine.

**Figure 7:**
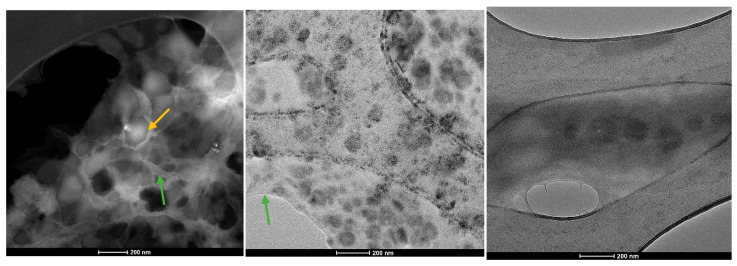
TEM micrographs obtained for DPPC_HMB_20mg, DPPC_NMN_20mg, and DPPC_L-Leucine_20mg (green arrow—lipid matrix, yellow arrow—lipid vesicles).

The lipid vesicles were further examined using transmission electron microscopy (TEM) to provide additional confirmation of the morphology and size of the DPPC-based vesicles. Figure 7 displays the TEM micrographs for samples containing the intermediate drug concentration, specifically DPPC_HMB_20mg, DPPC_NMN_20mg, and DPPC_L-Leucine_20mg.

The analyzed samples reveal the presence of DPPC-based lipid vesicles characterized by spherical morphology and dimensional uniformity. Moreover, higher-resolution imaging confirms the earlier findings, showing that the average dimensions of these vesicles remain in the nanometric range.

**Figure 8:**
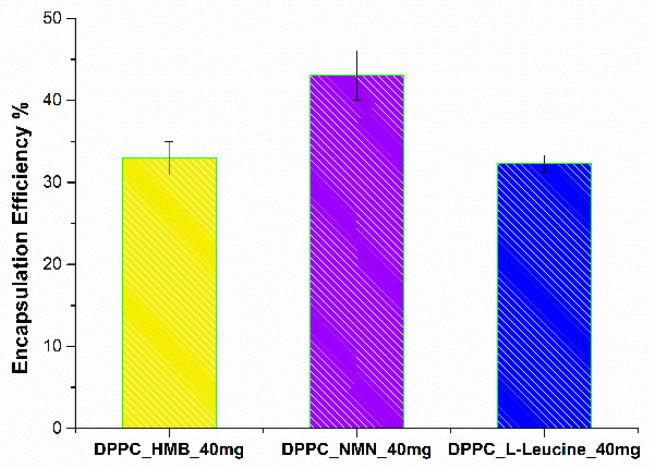
Encapsulation efficiency of HMB, NMN, and L-Leucine in DPPC_HMB_40mg, DPPC_NMN_40mg, and DPPC_L-Leucine_40mg.

**Figure 9:**
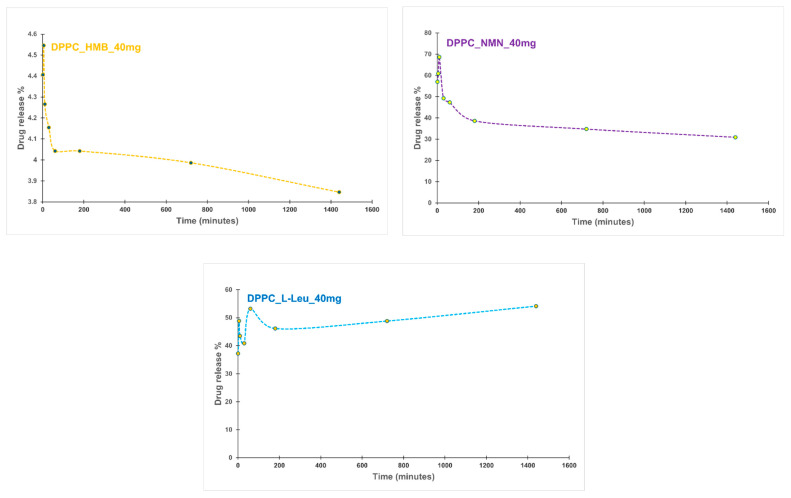
Drug release profile of DPPC_HMB_40mg, DPPC_NMN_40mg, and DPPC_L-Leucine_40mg.

The following section presents the results of the encapsulation efficiency (EE%) (Figure 8) estimation and drug release (Figure 9) analysis, providing insights into the performance of the formulated vesicles.

The EE% is influenced by the specific drug incorporated within the lipid vesicles. The measured EE% values were 32.95% for DPPC_HMB_40mg, 43.05% for DPPC_NMN_40mg, and 32.3% for DPPC_L-Leu_40mg. The comparable encapsulation efficiencies observed for HMB and L-Leucine may be attributed to their similar chemical characteristics, which likely influence their interactions with the lipid bilayer. Furthermore, the moderate EE% values recorded across all formulations could be associated with the limited amount of DPPC utilized in the formation of lipid vesicles, potentially constraining their encapsulation capacity.

The drug release kinetics from DPPC vesicles varied significantly depending on the encapsulated drug. For the DPPC_HMB_40mg sample, an initial burst release was observed in the first few minutes, followed by a gradual stabilization. The release decreased slightly from 4.40% at the first minute to 3.84% at 24 h, suggesting a controlled and sustained release. In contrast, DPPC_NMN_40mg showed a much higher initial burst release of 57.01%, which then declined gradually, stabilizing at 30.92% after 24 h. This also indicates a controlled diffusion mechanism. The rapid initial release suggests that NMN interactions with DPPC influence the release kinetic, leading to its rapid diffusion from the vesicles before a sustained release phase is established. DPPC_L-Leu_40mg also exhibited an initial burst release (37.28%), similar to the other formulations. However, this sample showed fluctuations in its release profile over time, potentially indicating vesicle instability or variable membrane permeability. Interestingly, the release increased to 54.14% after 24 h, yet maintained a slow and sustained release trend over time. The release profiles demonstrate that each drug exhibits a unique release pattern dictated by its molecular properties and interactions with the lipid bilayer. The initial burst effect, a common characteristic of drug-loaded lipid vesicles, was observed in all samples. The subsequent stabilization suggests a controlled release mechanism that could be beneficial for achieving prolonged therapeutic effects.

**Figure 10:**
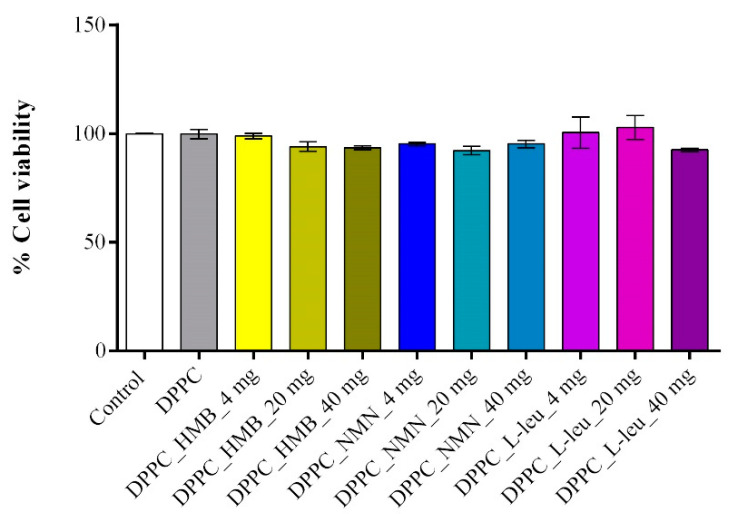
Graphical representation of C2C12 cell viability as revealed by the MTT assay after 24 h of treatment with DPPC lipid vesicles and drug-loaded formulations at various concentrations. Data are expressed as mean ± standard deviation (SD).

To determine the optimal working dose of both pristine and drug-loaded DPPC lipid vesicles for subsequent in vitro experiments, an MTT assay-based screening was performed. The results (Figure 10) demonstrated that all tested conditions maintained high cell viability, similar to the experimental control, confirming that DPPC vesicles, whether unloaded or drug-loaded, do not exhibit cytotoxic effects in C2C12 cell cultures. Cells treated with unloaded DPPC particles showed no significant viability changes compared to the untreated control, reinforcing the biocompatibility of the lipid vesicles. Likewise, drug-loaded formulations did not negatively impact cell survival, irrespective of the drug concentration, as none of the tested conditions led to a statistically significant decrease in viability. These findings highlight the safety profile of the DPPC-based drug delivery system, ensuring that drug cargo concentration does not interfere with the metabolic activity of C2C12 cells. Based on these results, the 40 mg/mL formulations were selected for further experiments, as they represent the highest tested concentration that maintained cell viability while ensuring a sufficient drug load for biological efficacy in subsequent functional assays.

**Figure 11:**
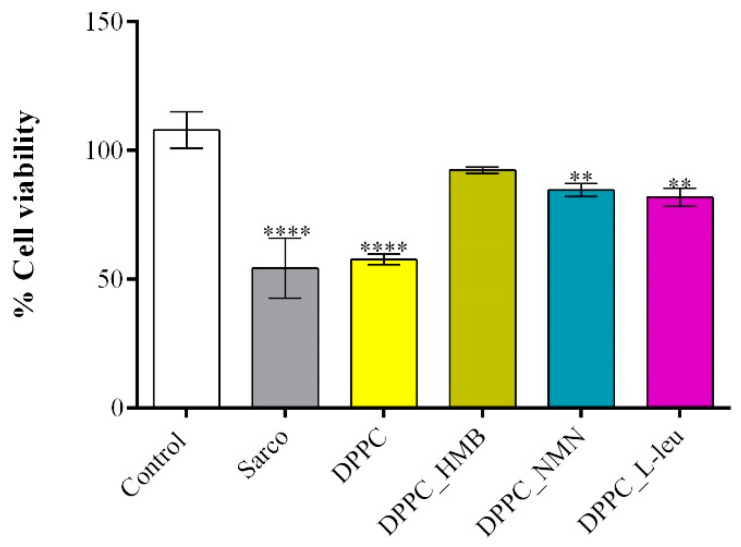
Graphical representation of C2C12 myotube viability after 48 h of treatment with simple DPPC particles and drug-loaded (HMB, NMN, L-Leu) lipid particles. Besides the experimental control, for all conditions, H_2_O_2_ was added to induce muscular atrophy in C2C12 myotube cultures. Data are expressed as mean ± standard deviation (SD). Statistical significance was determined using GraphPad Prism; *p*-values are indicated as follows: *p* ≤ 0.01 (**) and *p* ≤ 0.0001 (****) compared to the control group.

The C2C12 cell line was differentiated into myotubes, and H_2_O_2_ treatment was used to induce muscular atrophy, establishing an in vitro sarcopenia model. To evaluate the effects of lipid-based treatments on myotube viability after 48 h of exposure, an MTT assay was performed (Figure 11). Myotubes exposed to H_2_O_2_ exhibited a statistically significant reduction in cell viability compared to the experimental control, confirming that oxidative stress induced by H_2_O_2_ leads to substantial cellular damage and metabolic impairment. Similarly, cells treated with pristine DPPC vesicles displayed a comparable decrease in viability, indicating that DPPC alone does not provide protective effects against sarcopenia-induced stress. In contrast, drug-loaded DPPC formulations significantly improved cell viability compared to both the sarcopenic group and the DPPC-only group. Among these, DPPC-HMB demonstrated the strongest protective effect, restoring cell viability to levels comparable to the experimental control, suggesting that HMB effectively mitigates sarcopenia-induced cytotoxicity. DPPC-NMN and DPPC-L-Leucine also exhibited significant protective effects, though to a slightly lesser extent than HMB-loaded vesicles, as cell viability in these groups remained significantly lower than that of the experimental control.

**Figure 12:**
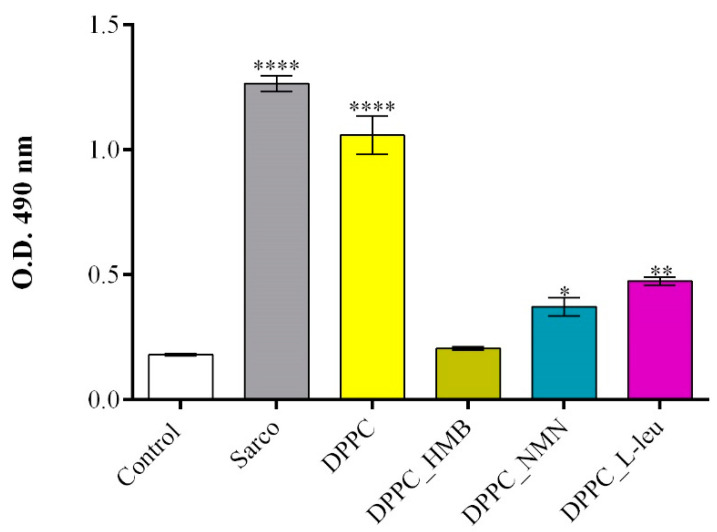
Graphical representation of LDH levels released in the culture medium by C2C12 myotubes after 48 h of treatment with simple DPPC particles and drug-loaded (HMB, NMN, L-Leu) lipid particles. Besides the experimental control, for all conditions, H_2_O_2_ was added to induce muscular atrophy in C2C12 myotube cultures. Data are expressed as mean ± standard deviation (SD). Statistical significance was determined using GraphPad Prism; *p*-values are indicated as follows: *p* ≤ 0.1 (*), *p* ≤ 0.01 (**) and *p* ≤ 0.0001 (****) compared to the control group.

The LDH assay was employed to assess the cytotoxicity of lipid-based treatments by measuring LDH activity in the culture supernatant, a marker of cell membrane integrity and damage. The results (Figure 12) were consistent with the findings from the MTT assay, further confirming the protective effects of drug-loaded lipid formulations. A significant increase in LDH release was observed in myotube cultures exposed to H_2_O_2_-induced oxidative stress, both in the absence and presence of pristine DPPC lipid vesicles, indicating severe membrane damage and loss of cell viability under these conditions. Notably, the DPPC-only group exhibited LDH levels comparable to those of the sarcopenic group, suggesting that in the absence of a drug cargo, DPPC lipid vesicles do not provide protection against oxidative stress-induced cytotoxicity. Conversely, drug-loaded DPPC formulations demonstrated a protective effect, with DPPC-HMB vesicles exhibiting the lowest LDH release, showing no significant increase compared to the untreated control. These results indicate that HMB effectively preserves membrane integrity and mitigates oxidative damage. Similarly, DPPC vesicles loaded with NMN and L-leucine also reduced LDH levels relative to the sarcopenic group, but to a lesser extent than DPPC_HMB treatment.

**Figure 13:**
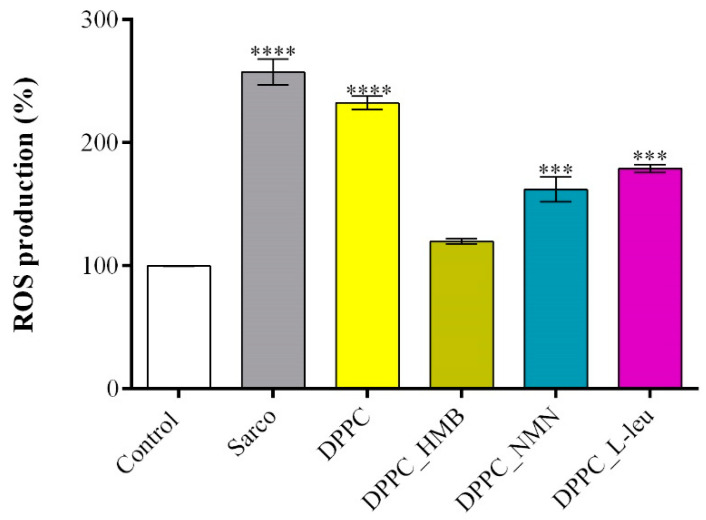
Graphical representation of ROS production in C2C12 myotube cell cultures after 48 h of treatment with simple DPPC particles and drug-loaded (HMB, NMN, L-Leu) lipid particles. Besides the experimental control, for all conditions, H_2_O_2_ was added to induce muscular atrophy in C2C12 myotubes culture. Data are expressed as mean ± standard deviation (SD). Statistical significance was determined using GraphPad Prism; *p*-values are indicated as follows: *p* ≤ 0.001 (***) and *p* ≤ 0.0001 (****) compared to the control group.

The evaluation of ROS production levels using 2′,7′-dichlorofluorescein diacetate (DCFH-DA) allowed the quantification of oxidative stress under the studied treatments (Figure 13). As expected, myotubes exposed to H_2_O_2_-induced oxidative stress exhibited a significant increase in ROS levels in comparison with the experimental control, oxidative stress being a peculiarity of sarcopenia. Similarly, pristine DPPC lipid vesicles failed to mitigate ROS accumulation, in this experimental group the ROS production level being comparable to that of the sarcopenic group. These findings indicate that DPPC alone does not provide antioxidant protection against H_2_O_2_-induced damage. In contrast, drug-loaded DPPC formulations effectively reduced ROS production, with DPPC_HMB demonstrating the strongest antioxidative effect. In myotube cultures exposed to DPPC_HMB treatment, the ROS levels were similar to the experimental control, suggesting its potential role in mitigating oxidative stress and preserving cellular redox homeostasis. Similarly, DPPC_NMN and DPPC_L-Leu treatments also decreased ROS levels, though their efficacy was lower than that of DPPC-HMB, as the ROS production was still significantly higher as compared with the experimental control.

**Figure 14:**
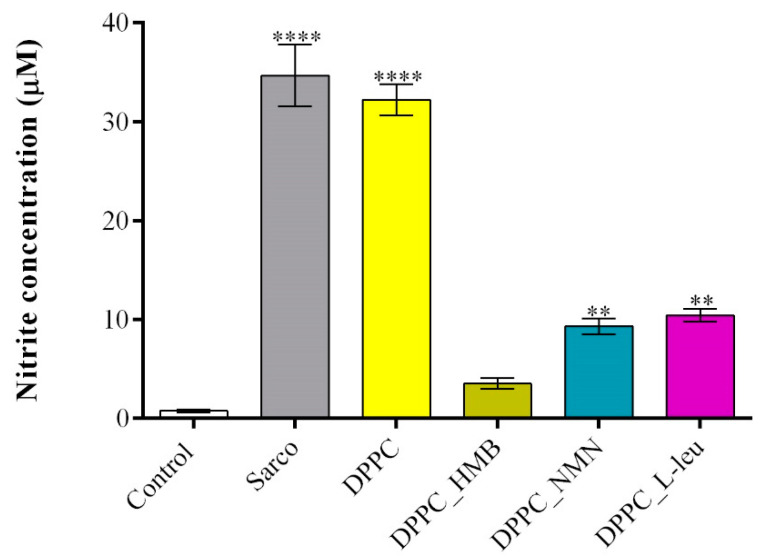
Graphical representation of NO production in C2C12 myotubes cell cultures after 48 h of treatment with simple DPPC particles and drug-loaded (HMB, NMN, L-Leu) lipid particles. Besides the experimental control, for all conditions, H_2_O_2_ was added to induce muscular atrophy in C2C12 myotubes culture. Data are expressed as mean ± standard deviation (SD). Statistical significance was determined using GraphPad Prism; *p*-values are indicated as follows: *p* ≤ 0.01 (**) and *p* ≤ 0.0001 (****) compared to the control group.

The quantification of the NO production (Figure 14) revealed that in the sarcopenia model (H_2_O_2_-treated myotubes), NO levels were significantly increased compared to the experimental control, confirming that oxidative stress triggers nitric oxide production, a hallmark of muscle atrophy and mitochondrial dysfunction. The addition of the pristine DPPC vesicles to the sarcopenia group did not significantly reduce NO levels, further reinforcing their lack of protective effects against oxidative stress-induced damage. In contrast, the drug-loaded DPPC formulations triggered a significant reduction in NO levels, with DPPC_HMB treatment showing the strongest effect, reducing NO production to near-control levels. While the efficacy of DPPC_NMN and DPPC_L-leu in reducing NO production was moderate compared to DPPC_HMB treatment, both demonstrated also protective effects as the NO levels were significantly lowered under both experimental conditions.

**Figure 15:**
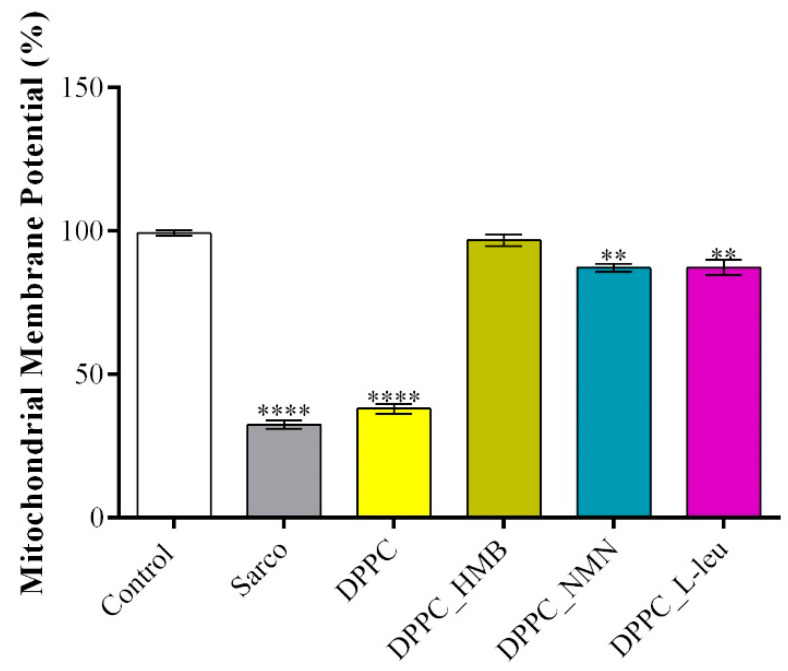
Graphical representation of MMP of C2C12 myotubes after 48 h of treatment with simple DPPC particles and drug-loaded (HMB, NMN, L-Leu) lipid particles. Besides the experimental control, for all conditions, H_2_O_2_ was added to induce muscular atrophy in C2C12 myotubes culture. Data are expressed as mean ± standard deviation (SD). Statistical significance was determined using GraphPad Prism; *p*-values are indicated as follows: *p* ≤ 0.01 (**) and *p* ≤ 0.0001 (****) compared to the control group.

The mitochondrial membrane potential (MMP) was assessed using a JC-10 fluorescence-based assay, a widely used indicator of mitochondrial integrity and function. The obtained results (Figure 15) revealed the extent of mitochondrial depolarization under oxidative stress of the tested treatments. As expected, myotubes exposed to H_2_O_2_-induced oxidative stress exhibited a significant decrease in MMP compared with the experimental control, confirming severe mitochondrial dysfunction and depolarization in response to H_2_O_2_-induced oxidative damage. Similarly, pristine DPPC lipid vesicles failed to prevent mitochondrial depolarization, as MMP levels remained significantly lower than the control group, indicating that DPPC alone does not provide mitochondrial protection under oxidative stress conditions. In contrast, drug-loaded DPPC formulations significantly restored MMP levels, suggesting their potential role in mitochondrial stabilization. Among the tested treatments, DPPC_HMB demonstrated the most pronounced protective effect, restoring MMP close to control levels. Similarly, DPPC_NMN, and DPPC_L-Leu also showed significant recovery in MMP, although their protective effects were less pronounced than those observed with HMB. This suggests that all drugs contribute to mitochondrial health as they act on mitigating mitochondrial damage in sarcopenia models.

**Figure 16:**
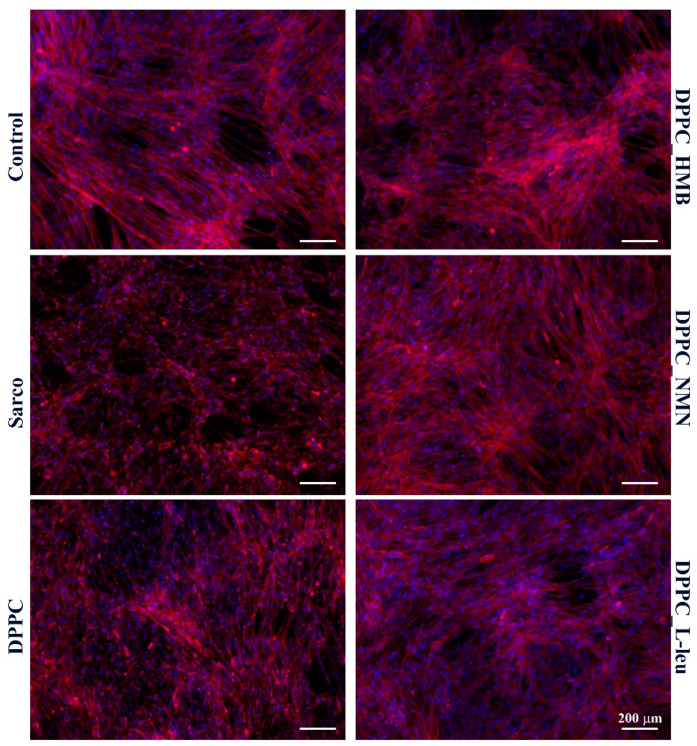
Fluorescence micrographs revealing F-actin filaments (red) and cell nuclei (blue) of C2C12 myotubes after 48 h of treatment with simple DPPC particles and drug-loaded (HMB, NMN, L-Leu) lipid particles. Besides the experimental control, for all conditions, H_2_O_2_ was added to induce muscular atrophy in C2C12 myotube cultures.

The cytoskeletal organization of C2C12 myotubes (Figure 16) was investigated by fluorescence microscopy after staining of F-actin filaments with TRITC-phalloidin. In the experimental control, myotubes displayed well-organized and aligned actin filaments with elongated and multinucleated structures, indicative of a healthy cytoskeletal network for muscle function. In contrast, in the sarcopenic group, a marked disorganization of actin filaments was observed, with loss of alignment, reduced filament density, and a fragmented cytoskeletal architecture, suggesting cytoskeletal degradation and impaired myotube integrity due to oxidative stress. The pristine DPPC formulation did not significantly improve cytoskeletal integrity, as actin filament disorganization remained evident, resembling the sarcopenic condition. This suggests that DPPC alone does not provide structural protection against oxidative stress-induced cytoskeletal damage. Conversely, the drug-loaded DPPC formulations (HMB, NMN, L-Leucine) improved actin filament integrity to varying extents. Among them, DPPC-HMB exhibited the most pronounced cytoskeletal restoration, with actin filament density and alignment closely resembling the control condition, indicating its protective role in maintaining myotube architecture and structural stability. DPPC-NMN and DPPC-L-Leucine also showed partial restoration of actin organization, although some degree of filament disorganization persisted, suggesting that while these formulations provide moderate protection, their effects on cytoskeletal integrity are less robust than those observed with HMB.

## 3. Discussion

This study aims to develop a novel therapeutic approach for treating sarcopenia through drug therapy designed to promote protein synthesis, enhance energy and glucose metabolism, diminish ROS levels, and stimulate the degree of nicotinamide adenine dinucleotide (NAD+). Lipid vesicles present an excellent option for this therapeutic application due to their properties that favor controlled drug release, as well as their superior biocompatibility. In this study, we utilized dipalmitoylphosphatidylcholine (DPPC), an amphiphilic lipid known for its capacity to spontaneously form vesicle-like structures in aqueous environments when stimulated by ultrasound. This characteristic makes DPPC particularly valuable for encapsulating therapeutic agents. To evaluate the efficacy of this approach, three different drugs—HMB (β-Hydroxy β-Methylbutyrate), NMN (Nicotinamide Mononucleotide), and L-Leucine—were individually encapsulated within DPPC-based vesicles. By conducting a comparative analysis of these formulations, we aim to assess their physicochemical properties and correlate these findings with their biological activity. This comprehensive evaluation will provide insights into the most effective strategy for mitigating sarcopenia through targeted drug delivery systems. Ultimately, the results of this study could lead to improved therapeutic interventions for individuals suffering from muscle degeneration, thereby enhancing their quality of life.

When developing lipid vesicles for drug encapsulation, a key objective is to produce spherical vesicles with uniform dimensions and stability in aqueous environments. The first analytical method employed was DLS, which is particularly well-suited for assessing materials with spherical morphology. We observed variations in the hydrodynamic dimensions of DPPC-based vesicles depending on the type of drug and its concentration. Specifically, both the drug type and concentration significantly influence the hydrodynamic diameter of the vesicles, which is also linked to the ultrasonication process. During ultrasonication, higher drug concentrations lead to increased fragmentation as more nuclei become available for vesicle formation. Consequently, raising the drug concentration while applying ultrasound results in the generation of smaller, more numerous lipid vesicles, which in turn reduces the hydrodynamic diameter [49,50].

Simultaneously, the DLS results, expressed in terms of zeta potential, indicate that DPPC vesicle-type liposomes exhibit stability in liquid form. Notably, as the concentration of the drug increases, the stability of the vesicle samples also improves, with optimal stability observed in DPPC liposomes containing the highest concentrations of HMB, NMN, and L-Leucine. This trend can be attributed to the relationship between hydrodynamic diameter and stability; specifically, vesicles forming with a smaller hydrodynamic diameter tend to exhibit greater stability. Among the three drugs examined, DPPC_NMN shows the most favorable zeta potential values, which can be attributed to the particulate nature of NMN. Its more complex molecular structure allows it to interact more effectively with phospholipids compared to HMB and L-Leucine. Furthermore, NMN may act as a potential co-stabilizer, containing polar groups that can interact favorably with the polar ends of DPPC phospholipids, enhancing the overall stability and interaction within the vesicle system [51,52,53,54,55].

All samples of DPPC vesicle-type liposomes exhibit a spherical morphology, as demonstrated by both SEM and TEM analyses. The vesicle edges display intact membranes with no discernible perforations in either lipid bilayer. The synthesis process significantly influences vesicle size, which falls within the nanometric range. The use of ultrasound in the formation and assembly of lipid vesicles contributes to the production of nano liposomes [56,57,58]. These findings are further validated by the correlations observed between the DLS results and the electron microscopy data.

The drug release rates of the samples were evaluated under time-dependent conditions, with a focus on the thermal sensitivity of DPPC lipid vesicles. The analysis was conducted at 40 °C, a temperature range (39–42 °C) within which DPPC exhibits elevated membrane permeability and facilitates drug release [59,60,61,62]. This thermosensitive property aligns with the targeted application for sarcopenia management, where localized heat application is used to alleviate musculoskeletal pain [63,64,65]. The release profiles exhibited distinct characteristics, primarily influenced by the type of drug encapsulated within the lipid vesicles. Notably, the DPPC_HMB_40mg sample showed the lowest percentages of drug release in the first 24 h, followed by DPPC_L-Leu_40mg and DPPC_NMN_40mg. A common feature among all three formulations was an initial burst release, a characteristic typical of lipid vesicles used for drug delivery [66,67,68]. While the release profiles for HMB- and NMN-containing vesicles stabilized over time, those encapsulating L-Leucine exhibited fluctuations, which may be attributed to vesicle instability. Nevertheless, the stabilization of the release profiles indicates a controlled release kinetics, which is advantageous for achieving prolonged therapeutic effects in the targeted application.

The biological investigations aimed to reveal the biocompatibility and therapeutic potential of DPPC lipid vesicles encapsulating HMB, NMN, and L-leucine in a H_2_O_2_-induced sarcopenia model by evaluating the potential of the treatments to counteract oxidative stress, mitochondrial dysfunction, and cytoskeletal degradation. Initial MTT-based screening confirmed the biocompatibility of both pristine and drug-loaded DPPC vesicles, demonstrating that none of the tested formulations exhibited cytotoxic effects on C2C12 myotubes, regardless of drug concentration. Based on these results, 40 mg/mL formulations were selected for further experiments to ensure optimal drug loading without compromising cell viability. Functional assays revealed that H_2_O_2_ exposure induced severe oxidative stress, leading to a significant decline in cell viability, increased LDH release, elevated ROS and NO production, and mitochondrial depolarization, confirming the establishment of a reproducible in vitro sarcopenia model [69]. While pristine DPPC vesicles failed to prevent oxidative damage, drug-loaded formulations exhibited substantial protective effects, with DPPC-HMB demonstrating the strongest efficacy in preserving cell viability, stabilizing mitochondrial function, reducing oxidative stress, and maintaining cytoskeletal integrity, consistent with its role in in mitochondrial biogenesis and oxidative stress modulation [36,70]. DPPC_NMN and DPPC_L-Leucine also showed protective effects, though less pronounced than HMB, suggesting differences in mechanisms of action, drug metabolism, or cellular uptake efficiency. These results collectively support the therapeutic potential of DPPC-based lipid vesicles as targeted drug delivery systems for sarcopenia management, with HMB-loaded vesicles emerging as the most effective formulation. Further investigations in in vivo models are warranted to fully elucidate the long-term benefits and mechanistic pathways underlying drug-loaded DPPC vesicles in muscle preservation.

## 4. Materials and Methods

The materials utilized in the formulation of lipid vesicles include 1,2-dipalmitoyl-sn-glycero-3-phosphocholine (DPPC), chloroform (CHCl_3_), distilled water, and three distinct compounds: beta-hydroxy beta-methylbutyrate (HMB), nicotinamide mononucleotide (NMN), and L-leucine. DPPC and chloroform were sourced from Avanti Polar Lipids, Inc. and Sigma-Aldrich (Burlington, MA, USA), while the three high-purity active ingredients were procured from a local supplement store, specifically for their potential to aid in muscle mass development and enhancement.

### 4.1. Synthesis of DPPC-Based Liposomal Vesicles and HMB, NMN, and L-Leucine Drug Encapsulation

To obtain DPPC liposomes, we began by dissolving DPPC in chloroform at 1:4 (*w*:*v*) (DPPC: CHCl_3_) in a round-bottom flask. This flask was placed in a rotary evaporator operating at 60 rpm under reduced pressure, with the water bath set to a temperature of 42 °C, which is required for the specific DPPC Tm [25]. The amount of DPPC, expressed in milligrams, was calculated so that each sample would contain 2 mg. Meanwhile, three aqueous solutions of the three drugs used were prepared, beginning with the first concentration in the study by adding 4 mg of HMB, NMN, and the corresponding L-Leucine, each in 20 mL of water and homogenizing until dissolved. The first synthesis series involves adding 10 mL of the DPPC stock solution to the aqueous drug solutions and exposing these mixtures to the ultrasound probe under the following working parameters: 20% amplitude for 5 min, alternating between 5 s on and 5 s off. The homogeneous solutions were gradually cooled to promote self-assembly. The turbidity of the mixture indicates that the process has commenced. Once this point was reached, the mixtures were kept at 25 °C for over 3 h to ensure the full incorporation of DPPC in the formation of micelles. The second series of syntheses involves the same process, but the concentration of each drug used is increased to 20 mg, and the third series of syntheses is also similar, with the difference of adding 40 mg of each individual drug. The same process was also performed for the control sample DPPC_Ctrl, but in the absence of any drug. Thus, 10 liquid samples of DPPC-based lipid vesicles were obtained, encapsulating different concentrations of HMB, NMN, and L-Leucine. Below is Table 4 that more simply expresses the content and code assigned to each sample obtained.

### 4.2. Analysis Methods

#### 4.2.1. Dynamic Light Scattering (DLS)

To evaluate two crucial characteristics in the production of lipid vesicles, we utilized the DelsaMax Pro from Beckman Coulter (Brea, CA, USA). This advanced equipment allowed us to monitor and record both the hydrodynamic diameter and zeta potential of each sample. The process began by injecting the prepared samples into the measurement cell of the DelsaMax Pro. Each sample was analyzed in triplicate to ensure the accuracy and reliability of the data collected. By doing so, we aimed to gain comprehensive insights into the size of the vesicles, their surface charge, and their stability in liquid media. Collectively, these measurements serve as key indicators of the physical properties of the lipid vesicles, guiding future modifications and optimizations in their formulation. By systematically analyzing these parameters, we can better understand how variations in lipid composition and drug encapsulation influence the overall behavior of the vesicles in therapeutic applications.

#### 4.2.2. Scanning Electron Microscopy (SEM)

Simultaneously, the physicochemical properties related to the morphology and physical size of lipid vesicles are critical parameters for defining their characteristics. To investigate these properties in detail, we employed a scanning electron microscope (SEM) model Inspect F50, which is equipped with an energy-dispersive spectrometer (EDS) from Thermo Fisher, FEI (Eindhoven, The Netherlands). For the analysis, 10 µL of each sample was carefully placed as a drop onto a carbon strip measuring 0.5 cm by 0.5 cm. The drops were allowed to dry at room temperature, ensuring that the integrity of the lipid vesicles was preserved during this process. Once dried, the samples stab was transferred to metallization equipment, where a thin layer of gold was deposited over the samples. Following metallization, the samples stab was introduced into the analysis chamber of the microscope. The micrographs were acquired by detecting the secondary electron beam and the electron beam scattering at an accelerating voltage of 30 keV.

#### 4.2.3. Transmission Electron Microscopy (TEM)

To achieve higher resolution and further validate the findings from previous analyses, the samples were subjected to transmission electron microscopy (TEM). For this process, 10 µL of each sample was carefully placed onto a 400-mesh lacey carbon-coated copper grid. The samples were left to air-dry at room temperature, ensuring that they retained their structural integrity during the drying process. The TEM analysis was conducted using a high-resolution 80–200 Titan Themis transmission electron microscope, acquired from Thermo Fisher Scientific (Oregon, OR, USA). Through this high-resolution imaging, we aimed not only to confirm the morphology and size distributions observed in the scanning electron microscopy (SEM) studies but also to explore finer details (the well-defined spheres and the full membrane integration without any disruption) that may influence the functionality of the lipid vesicles.

#### 4.2.4. Encapsulation Efficiency (EE%)

This evaluation aimed to separate lipid vesicles from the unencapsulated drug to determine EE%. In this experiment, UV Vis analysis was approached, and a Thermo Fisher Scientific Evolution 300 double-beam spectrophotometer (Waltham, MA, USA) was used for all spectrophotometric analyses. The included VisionPro software (version 4.5.0) was used for data processing. To achieve this, equal volumes of samples were centrifuged at 8500 rpm for 2 h, and the supernatant containing the unencapsulated drug was collected for analysis. The quantification of the unencapsulated drug concentration was performed using a five-point calibration curve established at different concentrations, ranging between 0.067 and 0.67 mg/mL for HMB and L-Leucine, and between 0.018 and 0.2 mg/mL for NMN. Spectral measurements were conducted within the 190–350 nm wavelength range, with each drug exhibiting a characteristic maximum absorption wavelength: 230 nm for HMB, 200 nm for L-Leu, and 260 nm for NMN. The resulting calibration curves demonstrated strong linearity, with correlation coefficients (R² values) of 0.9998 for HMB, 0.9995 for L-Leu, and 0.9991 for NMN. Based on the spectral measurements, the encapsulation efficiency (EE%) of the lipid vesicles was calculated using the following formula:

$$EE\%=\left(\frac{C_{total}-C_{free}}{C_{total}}\right)\times 100$$

where *C_total_* represents the concentration of the initially added drug, and *C_free_* represents the concentration of the un-encapsulated drug.

#### 4.2.5. Drug Release%

After separating the lipid vesicles from the unencapsulated drugs, the pellet obtained from each of the three samples after centrifugation was redispersed in ultrapure water and maintained in a water bath at 40 °C for 24 h. Samples were collected at specific time intervals of 1, 5, 10, 30, 60, 180, 720, and 1440 min. To ensure consistency throughout the evaluation, the same volume of ultrapure water was added to the sample after each collection to maintain uniform parameters. For the assessment of drug release, UV-Vis analysis was performed using the same equipment and calibration curves as those applied for encapsulation efficiency determination. The spectral measurements provided data for establishing the drug release profile of the three tested formulations, which was calculated using the following formula:

$$Drug\;release\%=\left(\frac{C_{release}\;}{C_{encapsulated}}\right)\times 100$$

where *C_release_* represents the time-dependent released drug concentration, and *C_encapsulated_* represents the concentration of the encapsulated drug.

### 4.3. In Vitro Biological Evaluation of HMB, NMN and L-Leucine Drug-Loaded DPPC Lipid Vesicles

#### 4.3.1. Cell Culture and Treatments

The C2C12 myoblastic cell line (ATCC CRL-1772) was used as an in vitro model for all biological investigations. Cells were maintained in Dulbecco’s Modified Eagle’s Medium (DMEM, Sigma-Aldrich), supplemented with 10% fetal bovine serum (FBS, Gibco, Thermo Fisher Scientific, Waltham, MA, USA) and 1% antibiotic-antimycotic solution (ABAM, Sigma-Aldrich). Cultures were incubated in a humidified atmosphere of 5% CO_2_ at 37 °C.

To establish the optimal working dose for lipid vesicles, C2C12 cells were treated with lipid vesicles loaded with varying concentrations of HMB, NMN, and L-leucine for 24 h. Cell viability was assessed using the MTT assay (3-(4,5-Dimethylthiazol-2-yl)-2,5-Diphenyltetrazolium Bromide, Sigma-Aldrich). Briefly, the culture medium was replaced with fresh MTT solution (1 mg/mL in DMEM) and incubated for 4 h to allow mitochondrial enzymatic reduction of MTT into insoluble formazan crystals. The formazan precipitate was then solubilized in isopropanol, and absorbance was measured at 550 nm using the FlexStation 3 microplate multimodal reader (Molecular Devices, San Jose, CA, USA). Cell viability was calculated as follows: Cell viability (%) = (Absorbance of treated cells/Absorbance of control cells) × 100.

Following initial viability assessments, C2C12 myoblasts were differentiated into myotubes for subsequent biological investigations. Cells were seeded in appropriate culture plates and allowed to reach 80% confluence. Differentiation was induced by serum deprivation, replacing the growth medium with high-glucose DMEM supplemented with 2% horse serum (HS, Sigma-Aldrich). Cells were maintained in a humidified atmosphere of 5% CO_2_ at 37 °C, and after four days of differentiation, myotubes were randomly divided into the following six experimental groups:

1. Control group—Differentiation medium refreshed without additional treatment.
2. Model group—Myotubes exposed to 100 μM H_2_O_2_ to induce a sarcopenia-like phenotype.
3. DPPC group—Myotubes treated with 100 μM H_2_O_2_ + DPPC lipid vesicles.
4. DPPC + HMB group—Myotubes treated with 100 μM H_2_O_2_ + DPPC lipid vesicles loaded with HMB.
5. DPPC + NMN group—Myotubes treated with 100 μM H_2_O_2_ + DPPC lipid vesicles loaded with NMN.
6. DPPC + L-Leucine group—Myotubes treated with 100 μM H_2_O_2_ + DPPC lipid vesicles loaded with L-Leucine.

All groups were cultured for 48 h under standard conditions, except for the control group, which received only fresh differentiation medium. Experimental groups (except control) were subjected to 100 μM H_2_O_2_ treatment to model sarcopenia, with or without the addition of drug-loaded lipid vesicles. Following 48 h of treatment, multiple biochemical and cellular assays were performed to assess mitochondrial function, cytotoxicity, oxidative stress, and cellular structure.

#### 4.3.2. Cell Viability Assessment

To investigate the impact of the lipid vesicle treatment on myotube cell viability, the MTT assay was performed as described above. Briefly, the culture medium was removed, and cells were incubated with 1 mg/mL MTT solution in fresh DMEM for 4 h at 37 °C. The resulting formazan crystals were solubilized in isopropanol, and absorbance was measured at 550 nm using the FlexStation 3 microplate reader (Molecular Devices). Cell viability was calculated as follows: Cell viability (%) = (Absorbance of treated cells/Absorbance of control cells) × 100.

#### 4.3.3. Cytotoxicity Assessment

The cytotoxicity of the lipid vesicles was measured based on the quantification of lactate dehydrogenase (LDH) release from damaged cells into the culture medium using the in vitro TOX7 LDH Cytotoxicity Assay Kit (Sigma Aldrich), following the manufacturer’s instructions. After 48 h of treatment, the culture supernatant was collected from each well and mixed with the LDH assay mixture, prepared as recommended in the kit. After a 30 min incubation step at room temperature in the dark, the enzymatic reaction was terminated by adding 50 μL of 1 N HCl. The absorbance was measured at 490 nm using the FlexStation 3 microplate reader.

#### 4.3.4. Reactive Oxygen Species (ROS) Measurement

Intracellular reactive oxygen species (ROS) levels were quantified using 2′, 7′-dichlorofluorescein diacetate (DCFH-DA, Sigma-Aldrich), a cell-permeable fluorescent probe that is non-fluorescent until oxidized by ROS into 2′,7′-dichlorofluorescein (DCF), a highly fluorescent compound. After 48 h of treatment, cells were washed with PBS and incubated with 10 μM DCFH-DA in serum-free DMEM for 30 min at 37 °C in the dark to prevent photooxidation. Following incubation, the excess dye was removed by washing with PBS, and fluorescence intensity was immediately measured at an excitation/emission = 485/530 nm using the FlexStation 3 microplate reader (Molecular Devices). ROS levels were expressed as a percentage relative to the control group, calculated as Ros Production (%) = (Fluorescence of treated cells/Fluorescence of control cells) × 100.

#### 4.3.5. Nitric Oxide (NO) Assay

Nitric oxide (NO) production was assessed using the Griess Reagent System (Promega) according to the manufacturer’s instructions. After 48 h of treatment, 50 μL of culture supernatant was collected from each well and transferred to a 96-well plate. An equal volume (50 μL) of Sulfanilamide Solution was added to each sample and incubated for 10 min at room temperature in the dark. Following this incubation, 50 μL of N-(1-Naphthyl)ethylenediamine Dihydrochloride (NED) Solution was added, and the plate was incubated for an additional 10 min. Absorbance was measured at 540 nm using a FlexStation 3 microplate reader (Molecular Devices). Nitrite concentrations were determined using a sodium nitrite standard curve ranging from 0 to 100 μM, and results were expressed as μM nitrite per sample.

#### 4.3.6. Mitochondrial Membrane Potential (MMP) Assessment

Mitochondrial membrane potential (MMP) was assessed using the Mitochondrial Membrane Potential Kit (MAK159, Sigma-Aldrich), which utilizes JC-10, a cationic, lipophilic dye that accumulates in mitochondria. After 48 h of treatment, 50 µL of JC-10 Dye Loading Solution (prepared by diluting 100× JC-10 in Assay Buffer A) was added to each well of a 96-well plate and incubated for 45 min at 37 °C in a 5% CO_2_ incubator, protected from light. Following incubation, 50 µL of Assay Buffer B was added to each well. Fluorescence was then measured using a FlexStation 3 microplate reader (Molecular Devices) at λex = 540 nm and λem = 590 nm for JC-10 aggregates (red fluorescence, polarized mitochondria), and λex = 490 nm and λem = 525 nm for JC-10 monomers (green fluorescence, depolarized mitochondria). MMP was expressed as the red-to-green fluorescence ratio, calculated as JC-10 ratio = Fluorescence at 590 nm (red)/Fluorescence at 525 nm (green).

#### 4.3.7. Cytoskeleton Investigation

To assess cytoskeletal integrity, F-actin filaments were stained with tetramethylrhodamine (TRITC)-conjugated phalloidin (Sigma Aldrich). Briefly, cells were fixed with 4% paraformaldehyde (PFA, Sigma-Aldrich) for 15 min and permeabilized using a blocking solution containing 2% bovine serum albumin (BSA, Sigma-Aldrich) and 0.1% Triton X-100 (Sigma-Aldrich) for 10 min at room temperature. Following fixation and permeabilization, cells were incubated with TRITC-phalloidin (1:100 dilution) for 1 h at 37 °C in the dark. Prior to imaging, cell nuclei were counterstained with 4′,6-diamidino-2-phenylindole (DAPI, Sigma-Aldrich) for 20 min to visualize nuclear morphology. Fluorescent images were acquired using an Olympus IX73 fluorescence microscope equipped with CellSense F software (V 8.0.2). Further image processing and quantification of cytoskeletal changes were performed using ImageJ (V 1.53, NIH, Bethesda, MD, USA).

#### 4.3.8. Statistical Analysis

All the biological experiments were performed in triplicate, and data were expressed as mean ± standard deviation (SD). Statistical analysis was conducted using GraphPad Prism V9 software, with the statistical significance threshold determined at *p* < 0.05.

## 5. Conclusions

This study presents a user-friendly, innovative pharmaceutical approach designed to address sarcopenia, a condition characterized by the degeneration of muscle tissue. Given the increasing prevalence of sarcopenia, particularly among the aging population, there is a critical need for effective interventions that are both easy to administer and capable of delivering meaningful results. To meet this challenge, we focused on the development of liposome-type lipid vesicles utilizing dipalmitoylphosphatidylcholine (DPPC) as a foundational component. This choice is particularly advantageous because DPPC lipid vesicles exhibit a membrane structure that closely resembles that of biological cell membranes, facilitating their interaction with the body while serving as an effective controlled drug delivery system. In this study, we selected three well-established drugs that have been previously tested and approved, particularly targeting consumers engaged in sports. These drugs not only demonstrate availability but also provide promising solutions for addressing the significant medical challenges posed by muscle degeneration. By employing lipid vesicles with nanometric dimensions, we successfully produced a stable liquid dispersion characterized by well-defined spherical morphologies and membrane integrity crucial for their functionality. Our investigations revealed a direct correlation between the concentration of the encapsulated drugs and the resulting performance of the lipid vesicles, particularly in terms of size and stability. Specifically, we observed that increased drug concentration resulted in smaller vesicle sizes, which, in turn, enhanced their stability. This finding is pivotal, as it suggests that meticulous optimization of drug loading can significantly influence the properties of the liposomal formulations, leading to improved therapeutic outcomes. With respect to the in vitro biological investigations, the obtained results highlight the strong therapeutic potential of drug-loaded DPPC lipid vesicles, particularly β-Hydroxy β-Methylbutyrate-loaded lipid vesicles, in mitigating oxidative stress, preserving mitochondrial membrane potential, and stabilizing cytoskeletal integrity in the H_2_O_2_-induced C2C12 myotubes sarcopenia model, making the original formulations promising candidates for further research in muscle degeneration therapies. From an academic and clinical research perspective, our findings underscore the intricate relationship between the properties of the material and the biological needs associated with sarcopenia. The synthesized and tested vesicles fulfill multiple criteria essential for promoting healing and mitigating the detrimental effects of skeletal muscle tissue degeneration. By addressing both mechanical and biochemical factors affecting muscle health, our research lays a solid foundation for developing effective therapeutic strategies aimed at combating sarcopenia.

Future studies will likely involve comprehensive biological evaluations of DPPC-based vesicles, focusing on in vitro cellular absorption and pharmacokinetic assessments by varying multiple parameters. In vivo evaluations directly on muscle tissue will also be a key area of investigation. These experiments will yield valuable insights that will inform choices regarding the routes of administration for the developed formulations, including options such as oral or injectable methods or potentially both. The ideal administration route will be one that balances patient comfort with therapeutic efficacy. Ultimately, the development of a clinically viable delivery system targeted at the elderly population is expected to support muscle regeneration, enhance mobility, and mitigate the effects of sarcopenia, a condition that is often overlooked due to the age of the patients. This novel therapeutic approach has the potential to significantly improve the quality of life for older adults affected by this debilitating condition.

## Figures and Tables

**Table 1:** Numeric values of hydrodynamic diameter (nm) as a DLS result of DPPC vesicles dispersed in water.

Hydrodynamic Diameter (nm)			
	4 mg	20 mg	40 mg
**DPPC_HMB**	243.76 ± 16.65	397.80 ± 16.30	153.80 ± 11.72
**DPPC_NMN**	112.46 ± 4.92	118.56 ± 12.20	235.60 ± 8.22
**DPPC_L-Leucine**	160.60 ± 5.6	245.16 ± 19.15	109.13 ± 7.74

**Table 2:** Numeric values of zeta potential (mV) as a DLS result of DPPC vesicles dispersed in water.

Zeta Potential (mV)			
	4 mg	20 mg	40 mg
**DPPC_HMB**	5.59 ± 0.63	6.90 ± 0.45	19.39 ± 1.17
**DPPC_NMN**	17.70 ± 0.43	26.99 ± 1.21	23.78 ± 0.94
**DPPC_L-Leucine**	−9.66 ± 1.33	−8.69 ± 0.84	−16.94 ± 1.71

**Table 3:** PDI values as a DLS result of DPPC vesicles dispersed in water.

PDI						
	4 mg	PDI Range	20 mg	PDI Range	40 mg	PDI Range
DPPC_HMB	0.081	<0.1 (Monodisperse)	0.011	<0.1 (Monodisperse)	0.081	<0.1 (Monodisperse)
DPPC_NMN	0.137	0.1–0.3 (Moderate)	0.102	0.1–0.3 (Moderate)	0.066	<0.1 (Monodisperse)
DPPC_L-Leucine	0.082	<0.1 (Monodisperse)	0.081	<0.1 (Monodisperse)	0.097	<0.1 (Monodisperse)

**Table 4:** Synthesis of DPPC-based vesicles and drug encapsulation process.

Synthesis Series					Follow the Process
DPPC stock solution	40 mL of H_2_O for hydrating the DPPC dried lipid film				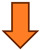
Drug aqueous solution	20 mL of H_2_O	4 mg/20 mg/40 mg of HMB in 20 mL of H_2_O	4 mg/20 mg/40 mg of NMN in 20 mL of H_2_O	4 mg/20 mg/40 mg of L-Leu in 20 mL of H_2_O	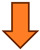
Mix of DPPC with drugs	10 mL DPPC solution + 20 mL of H_2_O	10 mL DPPC solution + HMB solution	10 mL DPPC solution + NMN solution	10 mL DPPC solution + L-Leucine solution	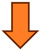
Sample code	DPPC_Ctrl	DPPC_HMB_4mg/20mg/40mg	DPPC_NMN_4mg/20mg/40mg	DPPC_L-Leucine_4mg/20mg/40mg	Finish

## Data Availability

The original contributions presented in this study are included in the article. Further inquiries can be directed to the corresponding authors.
